# Frontrunner in Translation: Progressive Supranuclear Palsy

**DOI:** 10.3389/fneur.2019.01125

**Published:** 2019-10-22

**Authors:** Ali Shoeibi, Nahid Olfati, Irene Litvan

**Affiliations:** ^1^Department of Neurology, Faculty of Medicine, Mashhad University of Medical Sciences, Mashhad, Iran; ^2^UC San Diego Department of Neurosciences, Parkinson and Other Movement Disorder Center, La Jolla, CA, United States

**Keywords:** progressive supranuclear palsy, tauopathy, translational research, epidemiology, etiopathogenesis, biomarker

## Abstract

Progressive supranuclear palsy (PSP) is a four-repeat tau proteinopathy. Abnormal tau deposition is not unique for PSP and is the basic pathologic finding in some other neurodegenerative disorders such as Alzheimer's disease (AD), age-related tauopathy, frontotemporal degeneration, corticobasal degeneration, and chronic traumatic encephalopathy. While AD research has mostly been focused on amyloid beta pathology until recently, PSP as a prototype of a primary tauopathy with high clinical-pathologic correlation and a rapid course is a crucial candidate for tau therapeutic research. Several novel approaches to slow disease progression are being developed. It is expected that the benefits of translational research in this disease will extend beyond the PSP population. This article reviews advances in the diagnosis, epidemiology, pathology, hypothesized etiopathogenesis, and biomarkers and disease-modifying therapeutic approaches of PSP that is leading it to become a frontrunner in translation.

## Introduction

**Figure 1:**
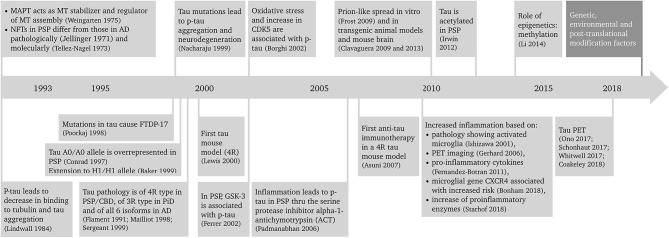
Twenty-five years of progress in progressive supranuclear palsy research. AD, Alzheimer's disease; CBD, corticobasal degeneration; CDK5, cyclin-dependent kinase 5; CXCR4, chemokine receptor type 4; FTDP-17, frontotemporal dementia with parkinsonism linked to chromosome 17; GSK-3, glycogen synthase kinase 3; MAPT, microtubule associated protein tau gene; MT, microtubule; NFT, neurofibrillary tangle; p-tau, phosphorylated tau; PET, positron emission tomography; PiD, Pick's disease; PSP, progressive supranuclear palsy; 3R, tau protein with 3 repeat domains; 4R, tau protein with 4 repeat domains.

Progressive supranuclear palsy (PSP) is a primary tauopathy that is playing an increasingly important role in the field. Better understanding of PSP clinicopathological correlations and pathogenesis has led to a revision of the diagnosis and search for new biomarkers and disease-modifying therapeutic approaches. Because of the rapidly progressive nature of the disease, PSP is an excellent candidate both for pre-clinical, animal model studies of tauopathies, and development of novel therapeutics that could be translated into the clinic. This article reviews the advances in the diagnosis, epidemiology, pathology, hypothesized etiopathogenesis, and biomarkers and disease-modifying therapeutic approaches of PSP that is leading it to become a frontrunner in translation. A summary of the progress in PSP research in the last 25 years is depicted in Figure 1 (1–27).

## Diagnosis

PSP was described as a clinicopathologic entity in 1964 by Steele et al. (28) who described nine patients with characteristic steady progressive vertical supranuclear gaze palsy, pseudobulbar palsy, cognitive impairment, retrocollis and axial rigidity. These features differentiated it from Parkinson's disease (PD) and encephalitic and vascular parkinsonism. They also mapped the neuronal degeneration characterized by neuronal cell loss, neurofibrillary tangles and gliosis in certain nuclei of the rostral midbrain, basal ganglia, and cerebellum (28).

Ten years later PSP was characterized as the prototype of subcortical dementias in view of its significant executive dysfunction and absence of cortical features (29). Early recurrent falls and slowing of vertical saccades were recognized later and were considered as the distinctive features of the National Institute of Neurological Disorders and Stroke and Society for PSP (NINDS-SPSP) possible clinical diagnostic inclusion criteria published in 1996 (30). The probable NINDS-SPSP criteria require both severe postural instability with falls within the first year of symptom onset and vertical supranuclear gaze palsy. These set of diagnostic criteria require lack of features excluding PSP such as cortical dementia resembling Alzheimer's disease (AD), autonomic disturbances or limb cerebellar features resembling multiple system atrophy, hallucinations, and delusions resembling dementia with Lewy bodies, lateralized cortical/motor features resembling corticobasal syndrome, oculomasticatory myoclonus characteristic of Whipple's disease and magnetic resonance imaging (MRI) abnormalities suggesting other disorders such as vascular parkinsonism, etc.

Pure akinesia with gait freezing was the first atypical PSP presentation that was reported by Japanese researchers in a number of pathologically diagnosed PSP cases in 1987 (31–33). Other PSP phenotypic presentations were later described in pathologically confirmed PSP patients (34), recognizing the PSP clinical heterogeneity. The original constellation of findings is now called PSP-Richardson syndrome (PSP-RS) or classical PSP (30).

The International Parkinson and Movement Disorder Society (MDS) has recently standardized the definition of several PSP phenotypic presentations (35). The new criteria (MDS-PSP) (35) classify the core PSP clinical features into four domains: ocular motor dysfunction, postural instability, akinesia, and cognitive dysfunction, which are classified into three levels of certainty (Table 1). Combinations of these features in different stages of the disease (especially early stages) determine the phenotype of PSP (35). In view of the considerable overlap between phenotypes (36), a set of four rules has recently been proposed by the MDS PSP study group to guide assignment of a unique phenotypical diagnosis to those patients who fulfill criteria for multiple phenotypes (37). However, it seems that still more refinement of the criteria is needed (38).

**Table 1:** Defining clinical features of the PSP phenotypes based on MDS-PSP criteria.

**Clinical domains**	**Certainty level**		
	**Level 1**	**Level 2**	**Level 3**
Ocular motor dysfunction	O1	O2	O3
	Vertical supranuclear gaze palsy	Slow vertical saccades	Macro square wave jerks or eyelid opening apraxia
Postural instability	P1	P2	P3
	Repeated unprovoked falls during the first 3 years of disease	Falls on pull-test during the first 3 years of disease	Three or more steps backwards on pull-test during the first 3 years of disease
Akinesia	A1	A2	A3
	Progressive gait freezing during the first 3 years of disease	Levodopa resistant bradykinesia with axial-dominant rigidity	Parkinsonism (bradykinesia and rigidity) with or without: tremor/asymmetry/levodopa response
Cognitive dysfunction	C1	C2	C3
	Speech/language disorders	Frontal cognitive/behavioral presentation	Corticobasal syndrome

*PSP-Parkinsonism* (PSP-P) is a retrospective diagnosis made after patients presenting with usually asymmetric parkinsonism with or without resting tremor or levodopa response resembling PD later develop typical PSP-RS features including postural instability and/or vertical supranuclear gaze palsy (34, 39). Williams et al. first defined PSP-P based on these features (34, 39), but the MDS criteria added an axial dominant parkinsonism (A2 in Table 1) as a more specific, though less sensitive, presentation for PSP-P (40).

*PSP-progressive gait freezing (PSP-PGF)* was originally described in Japan (32), and is currently defined as predominant transient motor blocks or start hesitations in the first 3 years of symptom onset which is unresponsive to levodopa and is unaccompanied with rigidity, tremor, or dementia early in disease course (35). Postural instability, falls and eye movement disturbances are late features of this variant and some patients never develop vertical supranuclear gaze palsy. Prominent axial and neck rigidity without limb rigidity are distinctive features for PSP-PGF. Conspicuous frontal subcortical dementia and levodopa responsive parkinsonism are not usual features (41). Patients with this phenotype, as well as the PSP-P phenotype have a longer survival than those with the PSP-RS.

A *frontal behavioral/cognitive syndrome* is the presenting feature of PSP in a small subset of patients (PSP-F) (42, 43). This phenotype is characterized by severe apathy, disinhibition, compulsive behavior and loss of insight. Typical PSP-RS features develop years later in the course of the disease. The MDS-PSP criteria defines frontal behavioral/cognitive syndrome as having two out of five basic cognitive features: apathy, bradyphrenia, dysexecutive syndrome, reduced phonemic verbal fluency, and socially inappropriate behaviors (impulsivity, disinhibition, or perseveration).

*PSP-non-fluent, agrammatic primary progressive aphasia/progressive apraxia of speech (PSP-nfaPPA/AOS), or the speech/language variant (PSP-SL)* is another cortical PSP variant (44) that is believed to have high specificity to represent a probable underlying 4R tauopathy pathology, along with the corticobasal syndrome (CBS) (35). *PSP-CBS* presents with a combination of cortical (ideomotor orobuccal or limb apraxia, parietal sensory dysfunction, and alien limb phenomenon) and movement (non-levodopa responsive akinesia, rigidity, and stimulus sensitive myoclonus) abnormalities. The standardized MDS definition requires presence of at least one feature of each, cortical or movement-related, categories. Sometimes these patients are clinically indistinguishable from patients with CBD-CBS, but the early presence of supranuclear vertical gaze palsy/slow vertical saccade or postural instability may favor the PSP-CBS diagnosis (45–48).

In the search for an early diagnosis of prodromal PSP, the MDS designates two variants: PSP-ocular motor (PSP-OM), defined as vertical supranuclear gaze palsy (O1), and PSP-postural instability (PSP-PI), defined as falls or postural instability on the pull test (P1 or P2). Both exclude other PSP clinical findings.

There are other rare variants described, which have yet to be standardized: PSP-primary lateral sclerosis (PSP-PLS) (49–51) and PSP-cerebellar ataxia (PSP-C) (52–54), but these are rare and have a very low predictive accuracy for PSP pathology (35).

Overall, the mean disease duration for PSP patient is about 6–8 years, with the shortest duration for PSP-RS (55). The main predictors of a short survival are the PSP-RS variant, early presence of falls, cognitive disorders, and dysphagia (56). Pneumonia and sepsis are considered as the leading causes of death in PSP patients (57).

## Epidemiology

PSP is the most common atypical parkinsonian disorder. It was considered as having an approximate prevalence of 5–6 per 100,000 (58, 59). However, higher PSP prevalence has been reported from Japan, Switzerland, and the United Kingdom (55, 60, 61). This higher prevalence could be related to the aging of population, increased general awareness of the condition, inclusion of various disease phenotypes and also the fact that recruitment occurred within a government-based program in Japan that provides support for rare disorders such as PSP. The fact that in some non-selected community-based brain autopsy series (62–64) 3–5% of cases with no or minimal clinical findings have showed PSP pathologies suggests that the true prevalence of PSP is probably much higher. PSP prevalence increases with age and shows no gender predominance (58, 59).

The cause of PSP is still unknown. The intriguing explanation of exposures to mitochondrial toxins as probable cause of PSP and the PSP-like Guadeloupean parkinsonism (65), as well as a cluster of PSP cases in an industrial region (66), suggests the role of environmental/toxic exposures in the PSP pathogenesis. A recent large incidence-based case-control study of 284 PSP and 284 matched controls (ENGENE-PSP) (67) showed that PSP is associated with lower education and exposure to well-water also supports those findings. The association of PSP with lower education is in accordance with previous studies (68, 69). The association with well-water consumption suggests pesticide exposure. Though pesticide, organic solvents, and metal exposures, assessed by an industrial hygienist and toxicologist, as well as living in or close to a rural area were significantly associated with PSP in univariate analyses, these factors were, however, not statistically significant in the multivariate analysis (67), which could be in part related to the relatively small study size. Note the possible contributory role of metal exposure found in the PSP cluster in Northern France (66) and the association between firearm use in veterans and PSP found in the ENGENE-PSP case-control study (70).

The epidemiological studies that assessed the PSP environmental risk factors are summarized in Table 2 (66–74). Available evidence collectively suggests a possible role of low education, metal exposure and consumption of well-water. It could be hypothesized that metal exposure and possible exposure to pesticides through well-water in people who work in those fields who may have low education could lead to increase in oxidative injury that in turn could lead to tau aggregation in susceptible individuals. However, it remains unclear why low education remains as an independent factor in the multivariate analyses. More research is needed to further identify the specific agent(s) responsible and possible mechanisms that would explain these observations (Table 2). Unfortunately only one of those studies had a relatively large sample to identify risk factors (67).

**Table 2:** Environmental epidemiological risk factors studies in PSP.

**References**	**Design/number of subjects**	**Region**	**Risk factor(s) assessed**	**Findings**
Kelley et al. (70)	Case-control/ 67 military veterans with PSP and 68 matched controls	North America	Firearm use as indicator of heavy metal (lead) exposure; Traumatic brain injury (TBI)	Firearm use was significantly higher in incident PSP cases vs. controls; Higher, but not significant, history of TBIs in incident PSP cases vs. controls
Park et al. (71)	Case-control/ 150 PSP women, 150 matched control women	North America	Low estrogen	Estrogen replacement therapy is associated with incident PSP cases, Inverse association of early menarche with PSP severity
Kelley et al. (72)	Case-control/ 76 PSP, 68 matched controls	North America	Lifetime stress exposure	Association of high-severity lifetime stressful events with incident PSP
Litvan et al. (67)	Case-control/ 284 PSP, 284 matched controls	North America	Environmental/Occupational	On univariate analysis: association of PSP incidence with lower income and education, and higher well-water use, years living in farm/near agricultural area, pack-years of smoking, years of transportation jobs and jobs with metal exposureOn multivariate analysis: association of PSP incidence with well-water use and inverse association with having a college degree
Caparros-Lefebvre et al. (66)	Report of a geographical PSP cluster/92 PSP	Northern France	Industrial exposure to chromate and phosphate ore processing; textile dyeing, and tanning	A cluster of Richardson and parkinsonism PSP phenotypes with an observed incidence of 12.3 times expected
Vidal et al. (69)	Case-control/ 79 PSP and 79 matched controls	France	Environmental/social/medical/toxic/family history	Low education level associated with PSP More frequent use of meat/poultry and less frequent use of fruits in PSP cases More frequent use of herbicides in PSP cases
Vanacore et al. (73)	Case-control/ 55 PSP, 134 matched controls	Italy	Smoking	No association of PSP prevalence with smoking
Golbe e al. (68)	Case-control/ 91 PSP (75 matched), 104 controls (75 matched)	North America	Environmental/ Occupational exposures Social/ medical/family history	Low education level associated with PSP prevalence
Davis et al. (74)	Case-control/ 50 PSP, 100 matched controls	North America	Viral, toxic, medical and surgical history Social and vascular risk factors	Living in areas with low population was associated with PSP prevalence No association of PSP prevalence with history of stroke, hypertension or smoking

## Pathology

Olszewski reported neurofibrillary tangles (NFTs) and gliosis in the basal ganglia (mainly globus pallidus and subthalamic nucleus), brainstem structures (predominantly superior colliculi, substantia nigra, periaqueductal gray matter, and pontine tegmentum), and cerebellar dentate nucleus in their cases (28). Pathological criteria of PSP, however, were developed about 30 years later (75) and subsequently revised and validated in 1996 (76) providing the basis for PSP research till present. It defines definite PSP as high density of NFTs and neuropil threads in at least three of these following areas: pallidum, subthalamic nucleus, substantia nigra, or pons. These changes should be accompanied by a pathology of low to high density in at least three of the following areas: striatum, medulla, oculomotor complex, or dentate nucleus. Fulfillment of these criteria in a patient with PSP-compatible history, after exclusion of ischemic and degenerative lesions diagnostic of other disorders, will define definite PSP. However, the tufted astrocyte which is characteristic of the disease was described a few years later by Komori et al. (77).

The establishment of the presence (78, 79) and central role of the tau protein in NFTs (8, 80, 81) and recognition of the six tau isoforms (82) was the key to the later identification of PSP as a 4R-tauopathy. This implies an over representation of the 4-repeat domain containing tau (4R-tau) isoforms relative to the 3-repeat containing ones (3R-tau) in its pathological tau aggregates (7, 83). This is in contrast to what occurs in healthy subjects that have both, 3- and 4-repeat tau isoforms in equal proportions. In PSP, hyperphosphorylated 4R-tau assembles into 13–14 nm straight filaments (84) that aggregate to form dense perikaryal “globose” NFTs in neurons and characteristic glial inclusions named “tufted astrocytes” (85, 86). It is hypothesized that PSP tau pathology in PSP-RS starts in the pallido-luyso-nigral areas and then spreads to the pontine nuclei, other basal ganglia structures, cerebellar dentate nucleus as well as frontal and parietal cortices. The various phenotypic presentations are a consequence of the pathology in different brain areas (87, 88).

In corticobasal degeneration, another 4-repeat tauopathy, neuronal NFTs are more disperse and less argyrophilic than in PSP and astrocytic plaques are the typical lesions because the aggregated tau is mainly located in cell processes, leaving cell soma almost devoid of aggregates. In contrast, PSP's tufted astrocytes are laden with tau fibrillar deposits at soma, with propagation to the cell processes (86, 89).

In contrast to these 4R tauopathies, tau pathology in AD mainly includes bundles of filaments that are composed of both 3R and 4R tau isoforms (90). These filaments are arranged in an antiparallel helical pattern as opposed to the straight filaments of PSP (91). AD tau aggregates mainly in neurons in special areas of the brain starting from transentorhinal regions with subsequent spread to the neocortical association areas (92).

## Genetics of PSP

**Figure 2:**
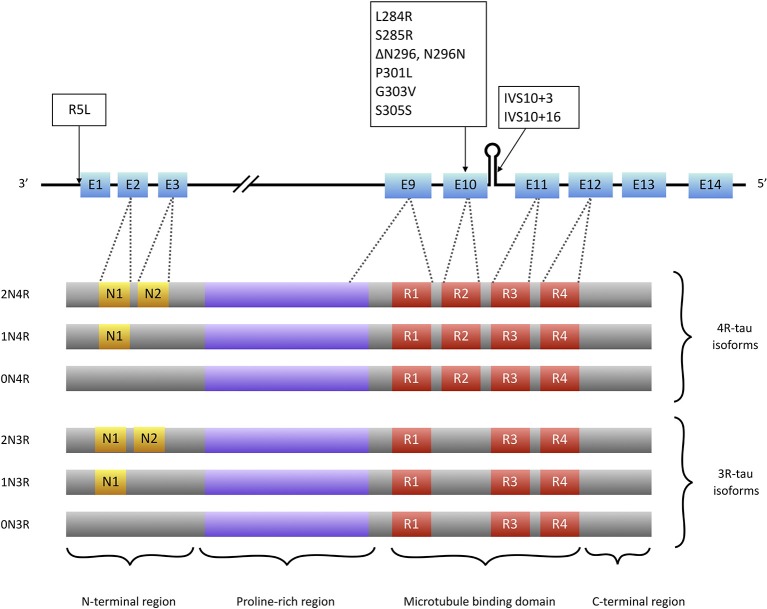
Tau isoforms and conformations. Six tau isoforms result from alternate splicing of exons 2, 3, and 10 (E2, E3, and E10). Tau mutations that present with PSP-like phenotypes (boxes) are mainly located at exon 10 or its splice site. There is only one mutation (R5L) located outside exon 10 that causes a PSP phenotype with brainstem 4R-tau aggregates but also 3R-tau-containing aggregates in cortical areas (93).

Tau is a microtubule associated protein coded by the MAPT (microtubule associated protein tau) gene located on chromosome 17q21. MAPT contains 16 exons. Alternate splicing of exons 2, 3 (inclusion of either 0, 1, or 2 near N-terminal inserts) and 10 [inclusion or exclusion of repeat-domain number 2 (R2)] produces all six tau isoforms (82) (Figure 2). Tau is extensively expressed in the brain and is mostly located in axons. It is believed traditionally that tau acts as a microtubule (MT) stabilizer and regulator of MTs assembly (94). Further studies showed that tau can regulate fast axonal transport (95) and probably plays a role in stabilizing nuclear DNA (96). To perform its functions, tau bears an intrinsically disordered conformation, meaning that it lacks a stable folded structure and this feature allows its interaction with a wide range of regulatory molecules (97, 98). Each tau isoform consists of 4 domains; an acidic N-terminus region containing a phosphatase-activation domain (PAD), a proline-rich domain, a MT binding region (with 3 or 4 repeat domains), and a neutral highly conserved C-terminal domain. In its monomeric water-soluble state, tau takes a paperclip conformation (99) that keeps the phosphatase-activation domain unexposed. The proline-rich domain includes much of the tau's potentially phosphorylatable regulatory sites, and the C-terminus region contains residues that probably prevent tau aggregation (100, 101).

The human MAPT gene is affected by a large inversion polymorphism that generates a region of linkage disequilibrium defined by two extended haplotypes, H1 and H2. The H1 haplotype is more frequent in PSP patients compared to the general population (9, 102). In fact, the H1 haplotype is present in 95% of the PSP patients compared to 75% of the general populations with an estimated odds ratio of 5.5, which indicates that H1 haplotype risk for PSP is the same to that of apolipoprotein E (APOE) ε3/ε4 risk allele for AD (103). A common variant of this haplotype, H1c sub-haplotype, has been shown to be accountable for the associated risk of the H1 haplotype with PSP and a number of other neurodegenerative disorders (104–106). A single nucleotide polymorphism (SNP) in the H1c background, rs242557, carries the major associated risk of this haplotype in PSP and CBD (107, 108). More recently three other H1 sub-haplotypes have been proposed to be associated with PSP including H1d, H1g, and H1o (109). The exact mechanism of pathogenesis of the H1 haplotype is not yet known, however there are some clues to confirm or denote to its role: (1) the PSP-like clinical phenotype has been observed in patients with familial tauopathy (frontotemporal degeneration with parkinsonism linked to chromosome 17, FTDP-17) who have an H1/H1 genotype in contrast to the frontal dementia-predominant phenotype in those with H1/H2 genotype (110), (2) increase in translation of total tau with a higher 4R:3R-tau ratio has been shown to be related to H1c sub-haplotype (111), (3) epigenetic studies have shown differential methylation pattern at this haplotype in PSP patients vs. controls (21, 112, 113), and (4) recently, increased plasma tau levels have been found to be associated with the H1c sub-haplotype (114). A MAPT variant, p.A152T, has also been found as a risk factor for PSP and other tauopathies (115).

Genome-wide association studies (GWAS) also found PSP-associated SNPs in other genes including STX6, EIF2AK3, and MOBP (104, 107). STX6 encodes the protein syntaxin 6 which is involved in protein trafficking through the endoplasmic reticulum (116). However, it is not yet known how this altered function would affect the tau metabolism in neurons and glia. (116) EIF2AK3 encodes an RNA-like pancreatic endoplasmic reticulum kinase (PERK), a regulator of the unfolded protein response (UPR) of this organelle. UPR is triggered when the endoplasmic reticulum is overloaded by unfolded proteins, and causes a reduction in overall protein translation and enhances autophagy (117). UPR is activated in regions of PSP brains involved by tau pathology (118). Studies on cultured neurons derived from PSP patients also showed that tauopathy-associated PERK alleles produce a functionally impaired kinase that is associated with neuronal damage due to endoplasmic reticulum stress (119). Product of the gene MOBP is a central nervous system (CNS) myelin structural protein highly expressed in the involved brain regions in PSP (120). Two recent GWAS studies with meta analyses (121, 122) revealed additional SNPs significantly associated with PSP inside or near three other genes: SLCO1A2, DUSP10, and RUNX2. SLCO1A2 codes for a solute carrier organic anion transporter that is highly expressed in areas commonly involved in PSP. A SNP found in an intergenic region near DUSP10 possibly has influence on tau hyperphosphorylation (122). RUNX2 codes for a transcription factor with an effect on differentiation of osteoblasts but its role in PSP is not yet known (121). These two large studies confirmed findings of previous studies except for an SNP in EIF2AK3 that did not reach genome-wide significance in one of these studies (121). Further studies are needed to confirm the latter findings and their implication in the PSP pathogenesis.

## Etiopathogenesis

It is not yet known which inciting event(s) trigger dysregulation of the tau protein and which tau abnormality precedes others in the sporadic PSP cases. The exact role of recent epidemiological studies described above need to be further studied (Table 2). A notion of possible contribution of tau “toxic gain of function” as the basic pathogenic underpinning of tauopathies was challenged after a study by SantaCruz et al. (123) who showed that switching off the abnormal expression of tau in a transgenic tauopathy mouse model stopped the neurodegeneration process and improved the cognitive function despite continued deposition of NFTs (124). Similar results were reported in other studies (125, 126). These findings indicate that the process of NFT formation could be dissociated from neurodegeneration and open a window for further discussion on the probability of existence of other more toxic soluble tau species that account for neurodegeneration (127, 128).

### Abnormal Post-translational Modifications

After translation, tau undergoes numerous regulatory post-translational modifications (PTMs) including phosphorylation, acetylation, methylation, truncation, among others. These PTMs can result in changes in tau conformation and in its affinity to MTs as well as its propensity to form aggregates (129). In the adult human brain, phosphorylation and dephosphorylation of tau by different kinases and phosphatases at various epitopes regulate the tau function, binding to microtubules and other membrane or nucleic acid partners, and axonal transport (95, 130). Tau contains 85 phosphorylatable epitopes (including serine, threonine, and tyrosine residues) but only 10 epitopes are phosphorylated in the normal brain compared to 16 epitopes in the PSP brains (131, 132). Abnormal tau phosphorylation is associated with a range of disturbances including: (1) tau detachment from MTs and impaired axonal transport (by unmasking of the PAD) (95, 101, 133, 134), (2) tau aggregation (especially through phosphorylation at the C-terminal region) (135), (3) redistribution of tau from axons to cell soma and dendritic processes causing impaired synaptic function and plasticity accompanied by α-amino-3-hydroxy-5-methylisoxazole-4-propionic acid (AMPA) and N-methyl-D-aspartate (NMDA) receptor rearrangements (136), and (4) impaired tau degradation by the proteasome via impairing its recognition by chaperons (137).

**Figure 3:**
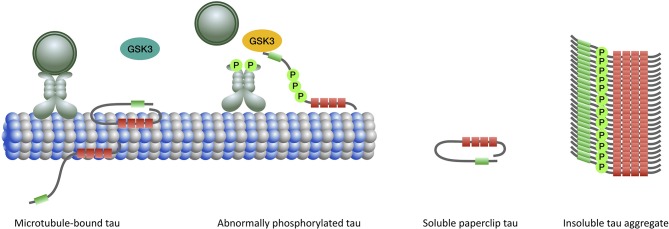
When tau binds to microtubules the N-terminal half of the protein projects away. The extreme N-terminal end of tau contains a phosphatase-activation domain (PAD) (green) that has a role in the regulation of cargo delivery. In the normal paperclip conformation PAD is not exposed, preventing it from triggering the phosphatase-kinase cascade (PKC) and detachment of cargo from microtubule. Activation of PAD and PKC normally occurs at the site of cargo delivery. Abnormal tau phosphorylation (by priming kinases) leads to persistent exposure and activation of PAD and triggering of the PKC which involves overactivation of GSK3β (yellow). Impairment of the fast axonal transport subsequently ensues. In the aggregated form, the microtubule binding domains constitute the core of the filament with the N- and C-terminal regions forming a fuzzy coat around it.

Considering the profound role of tau hyperphosphorylation in mediating various pathogenic processes leading to neurodegeneration in PSP, efforts have been mainly directed to elucidate the role of tau kinases and phosphatases. Tau is the substrate for a large number of kinases including proline-directed kinases, particularly glycogen synthase kinase 3 (GSK3) and cyclin-dependent kinase 5 (CDK5), many non-proline directed serine, and threonine kinases such as calcium/calmodulin-dependent protein kinase II, microtubule affinity-regulating kinases, cAMP-dependent protein kinase A) and tyrosine kinases (Fyn, Src, Abl) (101). GSK3 can phosphorylate almost half of tau's phosphorylatable sites and its abnormal activation has been shown in PSP as well as CBD and AD brains and is believed to be associated with pathologic tau hyperphosphorylation and aggregation (15). GSK3 is involved in a signaling pathway mediated by exposed PAD to trigger kinesin-bound cargo delivery. Abnormal exposure of PAD probably results in GSK3 activation and leads to cargo detachment from the MT as well as tau hyperphosphorylation and aggregation (95, 134) (Figure 3). However, the GSK3 inhibitors (including lithium, sodium valproate, and tideglusib) recently evaluated in clinical therapeutic trials of PSP patients failed to slow disease progression (138, 139). Other kinases and phosphatases are under evaluation (140). However, it is not yet known whether tau hyperphosphorylation is a cause for or a consequence of tau aggregation.

In addition, in PSP there is physiological and pathological acetylation on various lysine residues of the tau molecule (20). Acetylation of various residues provide stabilization of MT-bound tau and regulation of tau phosphorylation, aggregation, degradation, subcellular redistribution, truncation and tau liquid-liquid phase separation (20, 141–146). Acetylation at K280, which is located in the repeat domain 2 of the 4R tau, is a well-known pathological mechanism of acetylation in PSP and other tauopathies (20). However, further research is needed to reveal the acetylation profile of tau in PSP and evaluate its potential implication as a novel therapeutic strategy.

The addition of O-linked N-acetylglucosamine (O-GlcNAc) to the tau protein occurs physiologically and is believed to prevent tau hyperphosphorylation (147, 148). Although reduction of the enzyme involved in tau O-GlcNAcylation was shown in AD brains (149), there are no *in vivo* or *in vitro* studies in PSP.

Tau truncation can change its folding and this probably has effects on tau clearance leading to tau aggregation or other various toxic effects (150). Tau fragments enter the cerebrospinal fluid (CSF) and are being used as potential disease markers in many neurodegenerative diseases (151) including PSP. Other tau PTMs including nitration, ubiquitination, sumoylation, methylation, isomerization, and deamidation have mostly been studied in AD, but their role in PSP need to be clarified (101). A detailed profile of sequences and patterns of PSP tau PTMs in neurons and glia could increase our understanding of tau pathogenesis and provide new therapeutic targets.

### Mitochondrial Dysfunction

Several lines of evidence indicate the possible role of mitochondrial dysfunction, oxidative injury and defects of energy metabolism in PSP. Based on epidemiological and experimental studies, exposure to herbal neurotoxins containing mitochondrial complex I [CI, NADH:ubiquinone oxidoreductase (152)] inhibitors (mainly fruits and tea made from the Annonaceae family) is a risk factor for a PSP-like parkinsonian disorder with brainstem-predominant 4R-tau inclusions in the French West Indies (153–156). In addition, studies on PSP hybrid cell lines containing mitochondrial DNA showed CI hypofunction (157). PSP patients' brain positron emission tomography (PET) and phosphorous magnetic resonance spectroscopy provide further evidence of energy metabolism failure (158–160). Moreover, PSP brains studies show lipid peroxidation deficits and evidence of oxidative injury (161–163). Although these studies show possible energy metabolism defects in PSP, the exact biochemical basis for the mitochondrial dysfunction is not yet understood. Two recent small clinical trials of the coenzyme Q10 mitochondrial CI enhancer showed marginal or no benefit (164, 165).

### Neuroinflammation

Both direct postmortem examination of brain tissue and *in vivo* ligand-based PET studies show activation of brain macrophages and microglia in the PSP involved brain areas (13, 16, 19). Higher levels of proinflammatory cytokine transcripts, especially interleukin-1β, have also been reported in PSP brains (19) and a recent GWAS showed that the microglial gene CXCR4, is associated with increased risk of PSP and PD (26).

Other studies showed the role of the proinflammatory 5-lipoxygenase enzyme in PSP (26, 166). Despite all evidence for the role of neuroinflammation in the PSP pathogenesis, a PSP case-control study did not find any association between prior use of non-steroidal anti-inflammatory agents and PSP, its disease severity, or the age of symptom onset (167). However, the sample was not large enough to evaluate only anti-inflammatories that cross the blood-brain barrier.

### Prion-Like Tau Spread

Several studies show that in PSP abnormally phosphorylated tau fibrils act like self-propagating strains and produce pathogenic “seeds” that are transferable to neighboring cells and are capable of inducing tau aggregates in connecting neurons and glia following neural networks (168–171). However, it is not yet known which mechanisms in the cell-to-cell tau spread are the most relevant (exosomes, release and uptake, tunneling nanotubes, unconventional secretion, or other mechanisms) (172–175). Moreover, although studies are indicative of short-segment filamentous tau species as probable seeds (176, 177), the specific pathogenic PSP tau seeds and their conformation are still unknown. Detailed structure of the core of tau filaments derived from AD, Pick disease and chronic traumatic encephalopathy brains have recently been studied using cryo-electron microscopy (178–180). These studies provide interesting information about specific tau folding in AD, Pick's disease and chronic traumatic encephalopathy. Similar studies are being performed in PSP and corticobasal degeneration that will likely provide clues into the underlying tau pathogenic process, differentiate these disorders and equally important allow the modeling and development of new therapies based on the protein conformation.

## Diagnostic Biomarkers

There are no reliable biomarkers for the antemortem diagnosis of PSP. The diagnosis is currently based on clinical criteria. It is particularly challenging to differentiate PSP from a wide range of parkinsonian and dementing disorders during the first few years of disease in the absence of postural instability and ophthalmoparesis (181). Epidemiological studies show a lag of four or more years between the presentation of the first PSP symptom until the correct diagnosis (55, 58). Fortunately, due to increased PSP awareness this diagnosis lag is decreasing. Considering the lack of optimal biomarkers and the wide range of PSP phenotypic presentations that also overlap with other neurodegenerative proteinopathies PSP still remains underdiagnosed. Hopefully, ongoing studies focused on the characterization of PSP-specific biomarkers will soon identify accurate diagnostic and outcome biomarkers that would allow the conduction of therapeutic trials at earlier stages.

### Structural Brain Imaging

**Figure 4:**
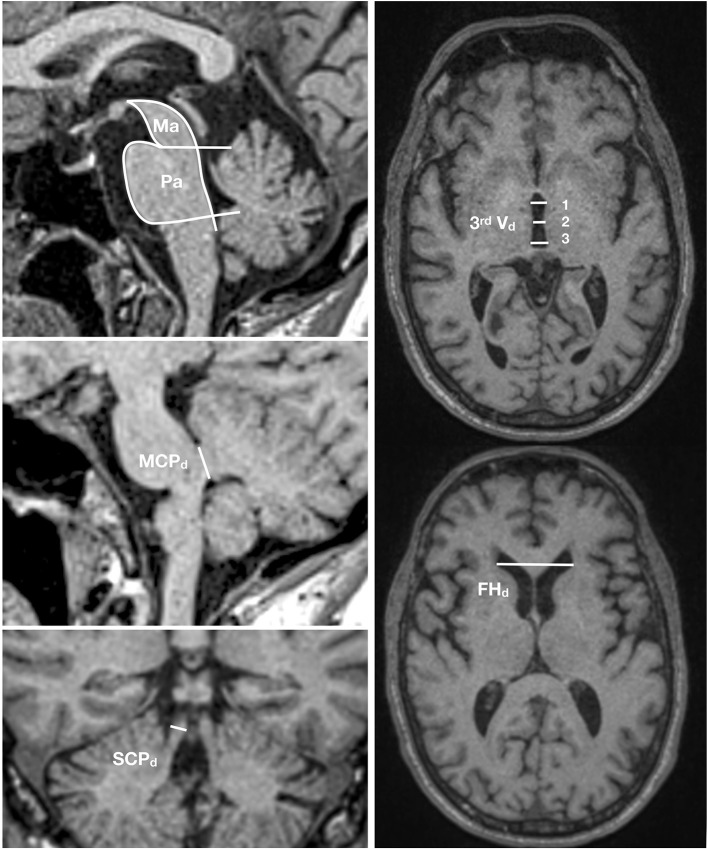
MRPI 2.0 index. This index is the product of the ratios of pons to midbrain area (Pa/Ma), width of middle to superior cerebellar peduncles (MCPd/SCPd), and average third ventricle diameter (measured at three points) to the maximal frontal horn diameter [(3rd Vd 1+2+3/3)/FHd]. MRIPI 2.0 = (Pa/Ma) × (MCPd/SCPd) × (average 3rd Vd/FHd).

Conventional brain imaging may show atrophy of midbrain and superior cerebellar peduncle out of proportion to that of the pons and middle cerebellar peduncle. Several imaging indices have been suggested as reliable markers of PSP-RS (182). However, imaging studies on pathologically confirmed PSP cases are limited and other conditions, especially CBD with clinical presentations of the Richardson syndrome, may have false positive indices (183). Therefore, the value of the MRI studies in increasing the certainty of the underlying pathologic diagnosis is unclear. The magnetic resonance parkinsonism index (MRPI) developed by Quattrone et al. (184) as the product of the ratios of pons to midbrain area (Pa/Ma) and middle to superior cerebellar peduncles diameter (MCPd/SCPd) showed a high sensitivity and specificity to differentiate PSP from PD and parkinsonism-predominant multiple system atrophy (MSA-P), another atypical parkinsonian disorder (184–187). A new variant of MRPI, named MRPI 2.0, that in addition incorporates the measurement of the third ventricle diameter (188) seems even more promising. MRPI 2.0 is defined as the MRPI ratio multiplied by the ratio of the third ventricle width of the frontal horn (Figure 4). However, pathologically confirmation in independent samples is still lacking.

Other structural imaging techniques such as voxel based morphometry, volumetry, diffusion weighted, and diffusion tensor imaging and combination of various measurements have been suggested to detect earlier stages of degeneration [for a review see (189)]. Lack of pathological confirmation in most studies as well as inherent limitations and confounders of these modalities are major shortcomings for these studies (190, 191). A number of small pathologically confirmed studies proposed that midbrain atrophy can differentiate PSP from other parkinsonian syndromes (192, 193). A larger pathologically confirmed study, using 3 dimensional MRI volumetry of the combination of midbrain, parietal white matter, temporal gray matter, brainstem, frontal white matter and pons, showed that this measure can reliably differentiate PSP from CBD and controls. However, a later study that differentiated typical and variant PSP phenotypes in their sample of 24 pathologically confirmed PSP showed that midbrain atrophy is associated with typical PSP phenotype (with underlying PSP or CBD pathology) but cannot differentiate PSP pathology presenting with variant phenotypes (183). Free water imaging (194) and diffusion kurtosis imaging (195) have recently been used to address parts of the limitations of structural imaging studies and have showed promising results. An increase in free water was found in several brain areas in PSP patients including basal ganglia, thalamus, midbrain, substantia nigra, cerebellar peduncles, dentate nucleus, cerebellar vermis and lobules V and VI, and corpus callosum. This pattern was in contrast to PD cases who had increased free water only in substantia nigra. MSA patients also had more restricted pattern than PSP cases (i.e., dentate nucleus, subthalamic nucleus, and corpus callosum did not show increased free water in MSA patients). Similarly, changes in free-water-corrected fractional anisotropy values were more pervasive in PSP (increased in putamen, caudate, thalamus, and vermis and decreased in the superior cerebellar peduncle and corpus callosum) than MSA (increased in putamen and caudate) and PD (no significant difference compared to controls) cases. These findings indicate that free water imaging might be used to differentiate various parkinsonian syndromes, however, replication of these findings in pathology proven samples is needed. These findings are in accordance with the results of the previous studies applying diffusion weighted and diffusion tensor imaging to parkinsonian patients (196–198) which in turn reflect the pattern of pathological involvement assessed by voxel based morphometry (199, 200). There are scarce studies focusing on differentiation between PSP phenotypes (201–204) but the results have been contradictory and inconclusive.

### Functional Imaging

Various functional imaging techniques have been used in parkinsonian syndromes including magnetic resonance spectroscopy, dopamine transporter imaging, task-free functional MRI, and FDG-PET (182). These techniques are not specific for the underling pathology and do not differentiate between neurodegenerative and non-degenerative processes.

Recently, tau PET imaging makes it possible to detect the distribution and severity of specific forms of tau pathology (182, 205). [_11_C]PBB3 (phenyl/pyridinyl-butadienyl-benzothiazole/benzothiazolium family), [_18_F]AV-1451 [aka [_18_F]flortaucipir, pyrido-indole family], and [_18_F]THK5351 (arylquinoline family) are the first generation of tau PET tracers tested in clinical studies (205). Unfortunately, [_11_C]PBB3 has off-target binding to white matter, venous structures and β amyloid (206) and [_18_F]AV-1451 has off-target binding to monoamine oxidase (MAO)-A, choroid plexus and mineralized or melanin containing structures (207). However, despite these off-target binding, studies on PSP patients showed that [_18_F]AV-1451 also binds to the PSP-specific subcortical areas with tau pathology, including dentate nucleus, thalamus, midbrain, pallidum and striatum (24, 25, 208, 209), which makes it a favorable tracer in studies of PSP. The pattern has been correlated with postmortem tau pathology in a few patients (24). In fact, [_18_F]AV-1451 has been reported to be highly sensitive and specific in differentiating PSP from PD (24). Nonetheless, off target binding precludes an early diagnosis because ligand binding is found in the same areas in normal controls except for the dentate nucleus (210–212). On the other hand, because these tracers were developed to detect 3R/4R AD pathology, the PET tau signal is lower in PSP, CBD and in patients carrying MAPT mutations with probable 4R tau pathology compared to those with AD (213). It remains controversial whether the tracer uptake associates with disease severity (208, 211). Despite the fact that postmortem PSP brain autoradiographic studies showed weak binding of this tracer to PSP pathology (207, 214), clinical studies show tau binding in PSP (24, 25, 208, 209). In fact, a recent study, evaluating disease progression in clinically diagnosed PSP-R patients followed up for 12 months using [_18_F]AV-1451 and midbrain volume on 3 Tesla MRI (215) showed that MRI midbrain atrophy correlated better with clinical disease progression than to [_18_F]AV-1451 uptake.

A recent study showed a correlation between PSP patients' clinical severity with the [_18_F]THK5351 signal in 11 PSP patients (216), however, there are concerns about its possible binding to TDP-43 pathology (217).

Second generation tracers including [_18_F]PM-PBB3, [_18_F]GTP-1 (ClinicalTrials.gov NCT02640092), [_18_F]PI-2620 (218, 219), [_18_F]MK-6240 (220), [_18_F]R06958948 (221), and [_18_F]JNJ64349311 (222) have generally showed less off-target binding especially at choroid plexus and MAO enzymes in AD and PSP. However, further validation in different samples including older patients are necessary. It is hoped that ongoing ligand studies identify 4R tau specific ligands. Detailed information of the PSP tau fibrillar structure once available will probably be helpful in designing more specific PSP tau ligands (223).

### CSF and Blood Biomarkers

Although high levels of tau oligomers in CSF have been well-incorporated into diagnostic workup of AD (224), tau oligomer measurements in PSP have not yet shown reliable and consistent pattern except for decreased level of total tau and phospho-tau compared to AD and healthy controls (225). It has been hypothesized that measurement of specific truncated forms or PSP-specific epitopes of tau released by degenerating cells in PSP are needed to show real amount of CSF tau in PSP. However, newer ELISAs with antibodies directed to mid- and N-terminal portions of tau showed the same results of lower tau levels in CSF (225). There are studies reporting a reverse association of CSF phospho-tau with disease severity in PSP (226).

Recently a protein amplification technique called real-time quaking-induced conversion (RT-QuIC) (227) or protein misfolding cyclic amplification (PMCA) assay (228), has been applied successfully in identification of small amounts of misfolded proteins in body tissue/fluid samples. RT-QuiC in CSF was recently used to discriminate PiD from other neurodegenerative disorders and healthy controls (229). The 3R-tau filaments of PiD, but not filaments from AD or FTD, seeded recombinant 3R tau monomers. 3R-tau RT-QuIC differentiated PiD from other disorders with high sensitivity and specificity. These results await replication in larger samples. The same method was used to differentiate AD tau seeds from disease controls including cases of 4R (PSP and CBD), 3R (PiD) or 3R+4R (chronic traumatic encephalopathy, primary age-related tauopathy) tauopathies (230). Results showed that AD-RT-QuIC assay can differentiate AD from 3R and 4R tauopathies with high sensitivity and specificity. These techniques are also under evaluation for possible application in identification of 4R-tau PSP seeds. However, at present, these techniques are not quantitative and do not allow to measure the disease severity.

Higher levels of neurofilament light chain (NfL) have been found in atypical parkinsonisms compared to PD (231). NfL is an unspecific marker of axonal loss in central and peripheral nervous system and studies on mouse models of tauopathy, AD, and α-synucleinopathy have showed that its blood and CSF levels are associated with progression and severity of neurodegeneration (232). NfL has been related to disease severity in PSP and changes in its level have been measurable in time-span of clinical trials, suggesting it could be a marker of disease progression in therapeutic trials (226, 233, 234). However, NfL is not suitable for PSP diagnosis since it can be high and associated with disease severity in other disorders such as vascular dementia and frontotemporal degeneration (235, 236). Various combinations of above-mentioned biomarkers have been proposed for the differential diagnosis of PSP, but replication studies are needed (226, 237, 238).

## Disease-modifying Therapeutic Approaches

Various disease modifying approaches are now under evaluation based on recent advances in the understanding of PSP pathogenesis (Table 3). Tideglusib, a GSK3-β inhibitor, was among the first disease-modifying agents evaluated in a large double-blind placebo-controlled clinical trial of PSP patients (138). Tideglusib failed to show any clinical effect as other GSK3-β inhibitors did in smaller trials (139). Davunetide, a neuroprotective and microtubule stabilizer, was evaluated with the same negative results (233). However, other phosphorylation inhibitors, microtubule stabilizers and neuroprotective agents are still under evaluation (140). Mitochondrial function enhancing nutrients including coenzyme Q10 have also been tested in clinical trials of PSP patients with no apparent benefit (140, 165).

**Table 3:** Disease modifying therapeutic approaches for PSP based on their target etiopathogenic process.

**Target**	**Reduction of abnormal PTMs**	**Blocking transcellular spread**	**Mitochondrial complex I enhancers**	**Autophagy enhancers**	**Microtubule stabilizers**	**Reduction of microglial activation and inflammation**	**Reduction of tau expression**
Preclinical/Hypothetical	• Novel GSK3β inhibitors • Inhibitors of other kinases • CDK5 • Brain specific calpain • ROCK	• Novel anti-4R-tau antibodies				• Benfotiamine (NRF2-dependent genes expression enhancer) • 5-Lipoxygenase blockers	• Antisense oligonucleotides • RNA interference silencing of tau expression
Ongoing clinical trials	• ASN120290 • MK-8719	• BIIB092		• AZP2006	• TPI-287		
Completed clinical trials	• Tideglusib • Sodium valproate • Lithium • Salsalate	• ABBV-8E12	• Coenzyme Q10 • α-lipolic acid with L-acetyl carnitine • Pyruvate with creatine and niacinamide	• Lithium	• Davunetide	• Salsalate	

Anti-tau antibodies are the most promising potential therapeutic strategies that are currently in clinical phase evaluation for PSP. Two humanized antibodies directed to different epitopes of extracellular tau, ABBV-8E12, and BIIB092, entered phase II with the hope to prevent the spread of tau pathology. ABBV-8E12 is a humanized antibody against extracellular fibrillar tau antibody designed to slow down the cell-to-cell spread of tau pathology. BIIB092 is directed to an N-terminally truncated form of extracellular tau. These agents showed no significant adverse events in the phase I trials and are being well-tolerated in ongoing phase II studies (140, 239). Unfortunately, ABBV-8E12 trial was recently discontinued due to lack of benefit.

MAPT gene silencing using antisense oligonucleotides or RNA interference are other promising future therapeutic strategies for tauopathies. IONIS-MAPT_Rx_ (BIIB080) is the only antisense oligonucleotide directed to the MAPT gene expression. It is currently under clinical evaluation in mild AD in a phase I/II study (ClinicalTrials.govNCT03186989) and is planned to enter phase II/III in FTLD patients.

## Conclusion

PSP is a pathological entity with a wide range of presenting clinical features. It may present with symptoms similar to other neurodegenerative disorders including other atypical parkinsonisms, PD, frontotemporal lobar degeneration, and AD. Recent advances in terms of phenotypic and pathologic characterization, genetics, and molecular imaging have greatly increased our understanding of this unique disorder and have provided clues for the development of disease modifying treatments. Further knowledge about the mechanisms involved in its development, pathological alteration at the level of genes, RNA, tau protein regulation and new insights into the structural details of 4R tau fibrils and seeds, will pave the way for novel therapeutic approaches. Development of 4R tau PET ligands and accurate measures of 4R-tau in blood are of utmost importance for an early diagnosis and measure of disease progression in new therapeutic trials.

Our better understanding of the etiopathogenesis is being translated into experimental therapeutic trials with anti-tau antibodies (240, 241) and a number of other therapeutic modalities, including antisense oligonucleotides (242), tau post-translational modifiers (138), neuroprotective (233), and anti-inflammatory drugs (243). Hopefully soon these new approaches will also translate into clinical practice.

## Disclosure

IL was supported by the National Institutes of Health grants: 5P50AG005131-33, 2R01AG038791-06A, U01NS090259, 1U54 NS 092089, U01NS100610, U01NS80818, R25NS098999, P20GM109025; Parkinson Study Group, Michael J Fox Foundation, Lewy Body Association, AVID Pharmaceuticals, Abbvie, Biogen and Roche. She was member of a Lundbeck Advisory Board and participated in a symposium organized by Sunovion. She receives her salary from the University of California, San Diego. She is Chief Editor of Frontiers in Neurology.

AS receives his salary from Mashhad University of Medical Sciences.

NO receives her salary from Mashhad University of Medical Sciences.

## Author Contributions

AS and NO prepared the primary draft of the manuscript and figures. IL supervised the prepration of the primary draft, reviewed the manuscript draft, and improved the text. All authors approved the final draft.

### Conflict of Interest

The authors declare that the research was conducted in the absence of any commercial or financial relationships that could be construed as a potential conflict of interest.

## Abbreviations

AD: Alzheimer's disease
APOE: apolipoprotein E
CBD: corticobasal degeneration
CBD-CBS: corticobasal degeneration with corticobasal syndrome phenotype
CI: mitochondrial complex I
CNS: central nervous system
CSF: cerebrospinal fluid
ENGENE-PSP: environmental genetic PSP risk factor study
FTDP-17: frontotemporal degeneration with parkinsonism linked to chromosome 17
FTLD: frontotemporal lobar degeneration
GSK3: glycogen synthase kinase 3
GWAS: Genome-wide association study
MAO-A: monoamine oxidase A
MAPT: microtubule associated protein tau gene
MDS-PSP: International Parkinson and Movement Disorders Society PSP study group criteria
MRI: magnetic resonance imaging
MRPI: Magnetic resonance parkinsonism index
MSA-P: parkinsonism-predominant multiple system atrophy ()
MT: microtubule
NfL: neurofilament light chain
NFTs: neurofibrillary tangles
NINDS-SPSP: National Institute of Neurological Disorders and Stroke and Society for PSP
O-GlcNAc: O-linked N-acetylglucosamine
PAD: phosphatase-activation domain
PD: Parkinson's disease
PERK: pancreatic endoplasmic reticulum kinase
PET: positron emission tomography
PiD: Pick's disease
PMCA: protein misfolding cyclic amplification
PSP: Progressive supranuclear palsy
PSP-C: PSP- cerebellar ataxia
PSP-CBS: PSP- corticobasal syndrome
PSP-F: PSP- behavioral variant of frontotemporal dementia
PSP-nfaPPA/AOS: PSP-non-fluent agrammatic primary progressive aphasia/progressive apraxia of speech
PSP-OM: PSP-ocular motor
PSP-P: PSP-Parkinsonism
PSP-PGF: PSP- progressive gait freezing
PSP-PI: PSP-postural instability
PSP-PLS: PSP- primary lateral sclerosis
PSP-RS: PSP-Richardson syndrome
PSP-SL: PSP- speech language variant
PTMs: post-translational modifications
R2: repeat domain number 2 of the tau gene
RT-QuIC: real-time quaking-induced conversion
SNPs: single nucleotide polymorphisms
UPR: unfolded protein response
3R-tau: tau containing 3 repeat domains
4R-tau: tau containing 4 repeat domains.
